# Autophagy and Human Parturition: Evaluation of LC3 Expression in Placenta from Spontaneous or Medically Induced Onset of Labor

**DOI:** 10.1155/2013/689768

**Published:** 2013-07-16

**Authors:** Laura Avagliano, Eleonora Virgili, Chiara Garò, Federica Quadrelli, Patrizia Doi, Michele Samaja, Gaetano Pietro Bulfamante, Anna Maria Marconi

**Affiliations:** ^1^Unit of Obstetrics and Gynecology, Department of Health Sciences, San Paolo Hospital Medical School, University of Milan, Via A. di Rudinì 8, 20142 Milan, Italy; ^2^Biochemistry and Molecular Biology Laboratories, Department of Health Sciences, San Paolo Hospital Medical School, University of Milan, Via A. di Rudinì 8, 20142 Milan, Italy; ^3^Unit of Human Pathology, Department of Health Sciences, San Paolo Hospital Medical School, University of Milan, Via A. di Rudinì 8, 20142 Milan, Italy

## Abstract

Induction of labor is one of the most used procedures in obstetrics, performed to achieve vaginal delivery through cervical ripening and stimulation of uterine contractions. We investigated the impact of induction of labor upon placental autophagy, a catabolic pathway activated in response to alteration of the physiological intracellular conditions. We collected 28 singleton placentas at the time of uncomplicated term vaginal delivery (7 spontaneous onset of labor, 21 induced labor). Autophagy was evaluated by immunohistochemistry, immunofluorescence, and immunoblotting. No significant difference in the autophagy expression was found between spontaneous or induced onset of labor. We found an inverse relationship between autophagy expression and the maternal prepregnancy body mass index, irrespective of the mode of labor onset. This result could be related to the nutritional maternal habits before and throughout pregnancy rather than rapid metabolic changes during labor.

## 1. Introduction

Autophagy is an inducible, intracellular catabolic pathway by which organelles or portion of cytoplasm is sequestered in autophagosomes, a double-membrane vesicle that fuses with lysosome to allow material breakdown and recycling [1].

In uncomplicated term pregnancies we have previously shown that autophagy is increased in placentas from cesarean when compared to vaginal delivery [2]; other studies have demonstrated higher levels of autophagy in pregnancies complicated by preeclampsia [3], intrauterine growth restriction [4–6], or both [4], when compared to normal pregnancies. In these studies placentas were collected at the time of elective cesarean section in both uncomplicated and complicated pregnancies.

Induction of labor is a procedure widely used in obstetrics, even though a number of common indications to induction have insufficient evidence to guide practice [7]. Nevertheless, as the procedure in the majority of cases leads to vaginal delivery, in the United States it has been estimated that approximately 1 in 4 women is inducted for maternal and/or fetal benefit [8]. Pharmacological induction of labor is an iatrogenic interruption of the uterine quiescence; autophagy is a process that responds to environmental changes and hormonal stimuli [9]. However, thus far, the impact of induction of labor upon placental autophagy has not been investigated even though placental autophagy itself is attracting the interest of researchers for its possible implications in maternal fetal medicine. For this reason, we underwent this study with the aim to investigate the relationship between autophagy and induction of labor; our hypothesis was that placental autophagy could be increased in cases of pharmacological induction; therefore, we evaluated the expression of autophagy markers in term placentas from vaginal deliveries after spontaneous or induced labor.

## 2. Materials and Methods

### 2.1. Cases Selection and Sample Collection

28 normal-shaped, singleton placentas were obtained at the time of uncomplicated term vaginal delivery from nonsmoking women with uneventful pregnancies. Seven placentas were from women with spontaneous onset of labor (group SP), while 21 placentas were collected from women with induction of labor performed according to the Bishop score: 7 cases with prostaglandin E2 only (group PG), 7 with oxytocin only (group OX), 7 with prostaglandin E2 followed by oxytocin (group PO). Cases selection was made matching patients for maternal and fetal characteristics: maternal age, prepregnancy body mass index, and neonatal birth weight were similar between the four groups.

No woman received medications during pregnancy and/or epidural analgesia during labor. 

In each woman we measured the time to delivery; umbilical arterial blood gases (pO_2_, pCO_2_), pH, base excess, lactate, and glucose concentration from a doubly clamped portion of the cord with a Radiometer ABL 700 analyzer; the weight of the placenta after trimming of the fetal membranes and umbilical cord and after removal of obvious blood clots; the longest diameter of the surface (*D*_1_) and its perpendicular diameter (*D*_2_), measured with a plastic ruler placed on the fetal surface; from these two measurements we calculated the placental surface area, assuming an elliptical surface, with the formula *D*_1_ × *D*_2_ × *π*/4. We also calculated the ratio between fetal and placental weight in grams, as the F/P ratio. 

Samples from grossly unremarkable placental parenchyma were collected immediately after delivery: full-thickness sections were selected and stored in 10% formalin solution for further immunohistochemical investigation; samples midway between the chorionic and basal plates were washed in phosphate-buffered saline solution to clear maternal blood, immediately frozen in liquid nitrogen, and stored at −80°C for further processing of protein extraction and Western blotting. Each placental section was sampled randomly in a site midway between the cord insertion and the periphery.

### 2.2. Investigated Markers

Placental autophagy expression was investigated utilizing *microtubule-associated protein light chain 3 (LC3)*. LC3 is the mammalian homologue of yeast Atg8 and intervenes in the late stage of autophagosome formation, particularly LC3-II, the membrane bound autophagic vesicle-associated form, that represents the phosphatidylethanolamine conjugated product of LC3-I that is obtained after LC3 activation [10]. For its role during the autophagosome genesis, LC3-II is commonly used as a specific marker of autophagy [11]. 

Placental *corticotropin-releasing hormone (CRF) *secretion is a marker of the timing of human parturition and delivery [12, 13]: placental expression of CRF and its relationship with LC3-II were investigated to detect any changes of autophagy expression related to placental hormonal changes in spontaneous or induced labor. 

Placental *hypoxia inducible factor (HIF) 1*α** is a transcription factor regulating the cellular response to hypoxia [14]: the expression of HIF-1*α* and its relationship with LC3-II were assessed to verify whether induction of labor might increase the level of placental hypoxia and, in turn, affect autophagy.

### 2.3. Immunohistochemistry

Immunohistochemical studies were carried out on 4 *μ*m thick tissue sections from formalin-fixed, paraffin-embedded tissue's samples, using a Novolink Polymer Detection System (Novocastra Laboratories) with primary rabbit polyclonal anti-LC3 antibody (NB100-2220, Novus Biologicals), rabbit polyclonal anti-CRF antibody (CRF, FL-196, Santa Cruz Biotechnology), and rabbit polyclonal anti-HIF-1*α* antibody (HIF-1*α*, H-206, Santa Cruz Biotechnology).

Sections were deparaffinized in Bio-Clear for 20 minutes then washed twice in ethanol. Antigen-retrieval bath containing 0.25 mM EDTA at pH 8 for 30 minutes at 95°C was used for CRF and HIF-1*α*, whereas a bath containing 9 mM sodium citrate at pH 6.0 for 30 min at 95°C was used for LC3. Endogenous peroxidase activity was quenched with 3% H_2_O_2_ in distilled H_2_O. Staining was performed with diaminobenzidine and fast red as a chromogen. For LC3, CRF, and HIF-1*α* staining, the primary antibody was applied at the dilutions of 1 : 500, 1 : 75, and 1 : 75, respectively and incubated overnight at 4°C. Slides with absence of the primary antibody were included as negative controls. Slides were immunostained in the same batch to ensure identical condition for comparison.

### 2.4. Immunofluorescence

Paraffin-embedded tissue sections were deparaffinized in xylene and rehydrated through a graded series of alcohols. LC3, CRF, HIF-1*α*, and cytokeratin 7 (CK7) were detected using rabbit polyclonal anti-LC3 antibody (NB100-2220, Novus Biologicals, dilution 1 : 500), rabbit polyclonal anti-CRF antibody (CRF, FL-196, Santa Cruz Biotechnology, dilution 1 : 100), rabbit polyclonal anti-HIF-1*α* antibody (HIF-1*α*, H-206, Santa Cruz Biotechnology, dilution 1 : 50), and mouse monoclonal anti-CK antibody (OV-TL 12/30, Dako, dilution 1 : 100), respectively. Slides were incubated overnight at 4°C. Fluorophore-conjugated secondary antibodies were employed as follows: polyclonal Swine Anti-Rabbit Immunoglobulins/FITC (F0205, Dako, dilution 1 : 50) for LC3 staining, polyclonal Swine Anti-Rabbit Immunoglobulins/TRITC (R0156, Dako, dilution 1 : 80) for CRF, HIF-1*α*, and polyclonal Rabbit Anti-Mouse Immunoglobulins/TRITC (R0270, Dako, dilution 1 : 100) for CK7 staining. Nuclei were subsequently counterstained with DAPI (4′,6-diamidino-2-phenylindole, Invitrogen). In the negative controls, the primary antibody was omitted and the specificity of the primary antibody was checked by using nonimmune rabbit IgG (Santa Cruz Biotechnology Inc.). Fluorescence images were viewed and captured using Imager.Z1 microscope (Zeiss).

### 2.5. Western Blotting

The 14000 ×g supernatant from homogenized samples was diluted with loading buffer, boiled, and stored at −20°C. 50 *μ*g of proteins were separated on 15% or 6% polyacrylamide gels (depending on the molecular weight of the markers studied) and transferred onto nitrocellulose.

LC3-I (cytosolic form, 18 kDa) and LC3-II (membrane-bound form, 16 kDa) were identified using primary rabbit monoclonal (LC3B antibody, Cell Signaling, dilution 1 : 1000). 

CRF (25 kDa) was identified using primary rabbit polyclonal antibody (CRF, FL-196 antibody, Santa Cruz Biotechnology, dilution 1 : 500).

HIF-1*α* (120 kDa) was identified using primary rabbit polyclonal antibody (HIF-1*α*, H-206X antibody, Santa Cruz Biotechnology, dilution 1 : 300). 

After washing, the blots were incubated with antirabbit horseradish peroxidase-conjugated secondary antibody (Jackson ImmunoResearch, dilution 1 : 10000). *α*-Tubulin was used for data normalization (*α*-tubulin (E-19)-R antibody, Santa Cruz Biotechnology, dilution 1 : 1000). Bands were visualized by LiteAblot reaction (EuroClone) and quantified (OD/mm^2^) by Quantity One 4.2.1 image analysis software (Bio-Rad).

### 2.6. Statistical Analysis

Clinical data are expressed as mean ± standard deviation (SD). Densitometric analysis of immunoblots is reported as mean and standard error of the mean (SEM). Clinical characteristics were compared by the Student's *t*-test for unpaired samples. The Kruskal-Wallis test was used to compare LC3-II, CRF, and HIF-1*α* protein levels in placental samples. *P* values <0.05 were considered significant. Statistical tests were performed using Instat 3, GraphPad software.

## 3. Results

### 3.1. Clinical Characteristics

Maternal age, BMI, gestational age at delivery, newborn and placental weight, placental surface, F/P ratio, umbilical arterial blood parameters, and time to vaginal delivery were similar in the three groups of women undergoing labor induction; thus, the data were pooled together and are presented in Table 1, compared to those obtained in women in spontaneous labor. As expected time to vaginal delivery was significantly shorter (*P* = 0.03) in women with spontaneous labor.

### 3.2. Autophagy Localization and Expression

**Figure 1:**
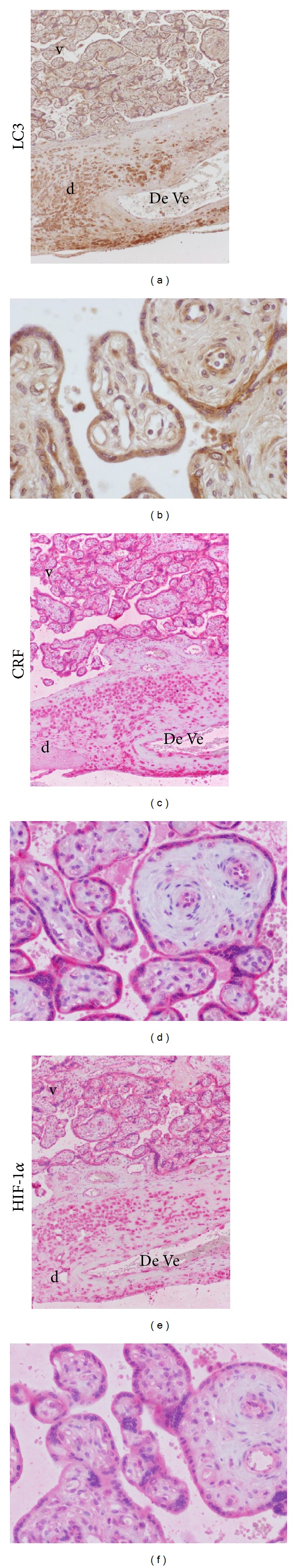
Localization of LC3, CRF, and HIF-1*α* by immunohistochemical method. The immunohistochemical staining shows that LC3, CRF, and HIF-1*α* have an overlapping localization in villous and extravillous trophoblast. d: decidua; v: villi; DeVe: decidual vessel. ((a) and (b) Immunohistochemical LC3 expression, original magnification 10x and 40x, respectively; (c) and (d) Immunohistochemical CRF expression, original magnification 10x and 40x, respectively; (e) and (f) Immunohistochemical HIF-1*α* staining, original magnification 10x and 40x, resp.) Images obtained from SP case.

**Figure 2:**
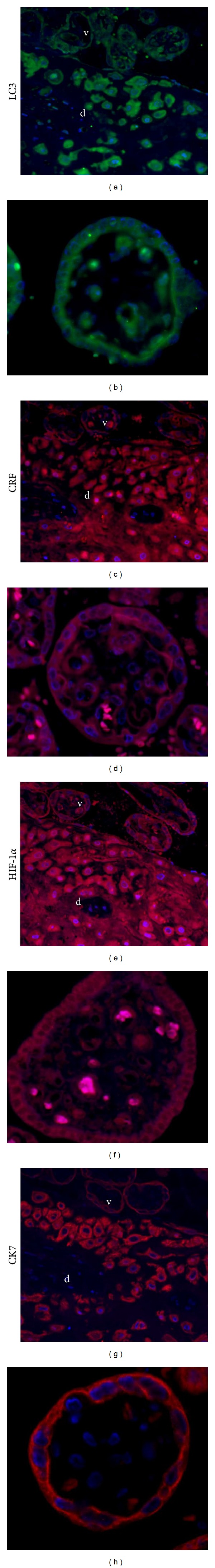
Localization of LC3, CRF, and HIF-1*α* by immunofluorescence method. Immunofluorescence method confirms the localization of LC3, CRF, and HIF-1*α* in villous and extravillous trophoblasts. Cytokeratin 7 was used as a marker of trophoblastic cells. d: decidua; v: villi. ((a) LC3 expression in trophoblastic cells in the decidual layer, original magnification 20x; (b) LC3 expression in villous trophoblast, original magnification 40x; (c) CRF expression in trophoblastic cells in the decidual layer, original magnification 20x; (d) CRF expression in villous trophoblast, original magnification 40x; (e) HIF-1*α* expression in trophoblastic cells in the decidual layer, original magnification 20x; (f) HIF-1*α* expression in villous trophoblast, original magnification 40x; (g) CK7 expression in trophoblastic cells in the decidual layer, original magnification 20x; (h) CK7 expression in villous trophoblast, original magnification 40x.) Images obtained from SP case.

By immunohistochemistry we detected cytoplasmic staining of LC3 in the amniotic epithelium, in the villous vessels, in villous syncytio- and cytotrophoblasts, in decidual stromal cells, and in extravillous trophoblasts (Figure 1). This localization of the autophagic marker was confirmed by immunofluorescence method, by detecting LC3 dot formation (Figure 2).

**Figure 3:**
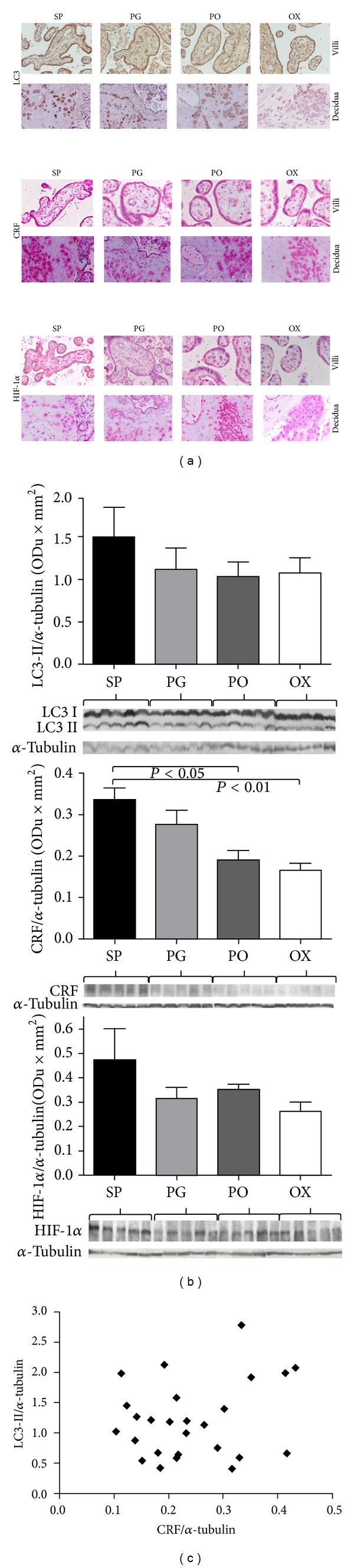
LC3, CRF, and HIF-1*α* expression. (a) for each antibody the spatial distribution of immunohistochemical staining was the same in cases with spontaneous and induced labor. (b) The analysis of data from optical density values of Western blotting bands shows no differences of LC3-II and HIF-1*α* expression between groups. On the contrary CRF is higher in spontaneous labor. LC3-II, HIF-1*α*, and CRF are normalized onto *α*-tubulin. (c) No correlation between LC3-II and CRF expression was found. SP: spontaneous labor; PG: induction with prostaglandin; PO: induction with prostaglandin and oxytocin; OX: induction with oxytocin.

The spatial distribution of staining was the same in cases with spontaneous and induced labor (Figure 3).

Figure 3 shows the expression of LC3-II according to the mode of the onset of labor: no significant difference was found among groups. 

### 3.3. Correlation between Autophagy and CRF and HIF-1*α*

The pattern of immunostaining for CRF and HIF-1*α* was the same that for LC3 (Figures 1 and 2) but not the intensity: HIF-1*α* staining was weaker than LC3 and CRF staining.

Figure 3 shows the expression of CRF and HIF-1*α* according to the mode of labor onset. Similarl to LC3-II, HIF-1*α* expression was not significantly different between any of the groups. On the contrary, as expected, a significantly higher expression of CRF was detected in placentas from spontaneous onset of labor; however, we found no significant relationship between the levels of CRF and LC3-II (Figure 3).

### 3.4. Correlation between Clinical Characteristics and Autophagy Expression

**Figure 4:**
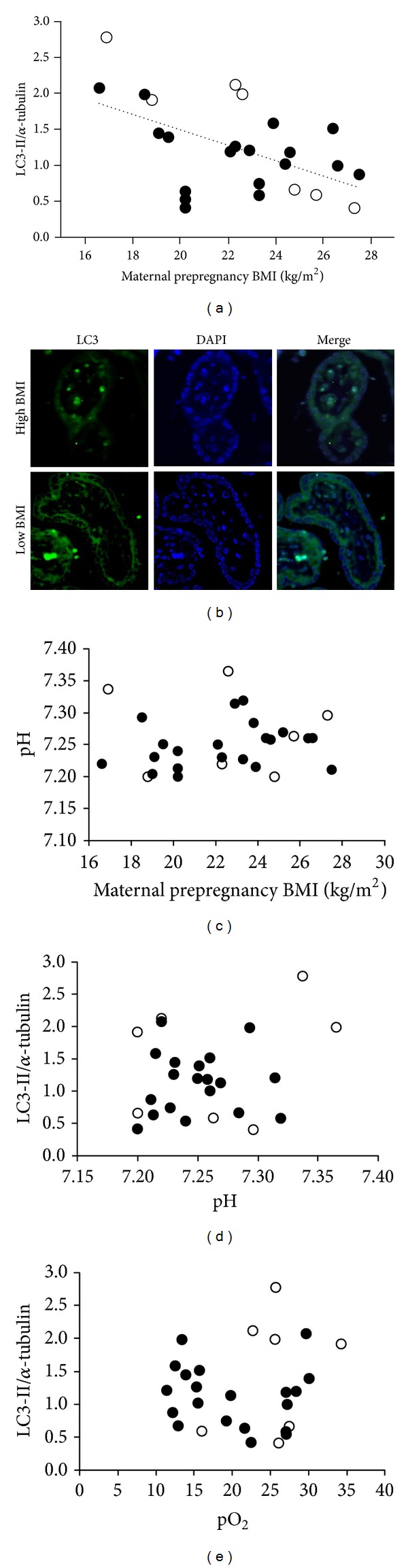
Autophagy and clinical correlation. (a) A significant correlation was found between LC3-II expression and prepregnancy maternal body mass index. Open circles: spontaneous onset of labor; closed circles: pharmacological induction of labor. LC3-II = 3.6–0.11 BMI. *R* ^2^ 0.29. *P* = 0.003. (b) LC3 dots formation identified by immunofluorescence in two cases of our population. Lower panel: PG group, patient with BMI = 16.6 kg/m^2^, LC3-II/*α* tubulin = 2.07, and umbilical cord pH = 7.22. Upper panel: PG group, patient with BMI = 26.6 kg/m^2^, LC3-II/*α* tubulin = 1.0, and umbilical cord pH = 7.25. (c) No relationship was found between maternal pre-pregnancy BMI and pH in umbilical cord. Open circles: spontaneous onset of labor; closed circles: pharmacological induction of labor. (d) No relationship was found between autophagy and pH in umbilical cord. Open circles: spontaneous onset of labor; closed circles: pharmacological induction of labor. (e) No relationship was found between autophagy and pO_2_ (mmHg) in umbilical cord. Open circles: spontaneous onset of labor; closed circles: pharmacological induction of labor. BMI: body mass index.

We found no relationship between LC3-II expression and any of the clinical parameters (time to vaginal delivery and maternal and fetal characteristics) with the exception of pre-pregnancy BMI: as BMI increased, placental autophagy expression decreased (*P* < 0.005, Figure 4). To verify if this result could depend on a hypoxic status present in pregnant woman of lean habit, we evaluate the correlation between maternal pre-pregnancy BMI and pH in umbilical cord at birth, but no relationship was found (Figure 4); furthermore, no relationship was found between placental LC3-II expression and pH or pO_2_ in umbilical cord (Figure 4).

## 4. Discussion

Autophagy is a Greek term coined by Christian DeDuve to indicate “self-eating” [15]. During the autophagic process, macromolecules (such as sugars, proteins, and lipids) and organelles (such as mitochondria) are degraded by lysosome to warrant a cellular adaptive response during compromised conditions [16]. In our previous work, in uneventful term pregnancies, we demonstrated a higher autophagy expression in placentas obtained from cesarean section than from vaginal delivery [2]. This suggested that the mode of delivery *per se*, or any other factor linked to it, such as fasting before cesarean section, could affect the expression of the markers of autophagy. In other words, the presence of a healthy mother and newborn at the end of an uneventful pregnancy is not sufficient to consider the level of autophagy in placenta as a “basal” levels and the mode of delivery should be taken into account when comparing placentas from normal and abnormal pregnancies. For this reason we were interested in investigating the influence, if any, of the mode of onset of labor on autophagy expression.

According to expectation [17], placental CRF expression in induced labor was lower than in spontaneous onset; however, no significant relationship was found with LC3-II expression. 

Pharmacological induction of labor aims to achieve vaginal delivery through the processes of cervical ripening and onset of uterine contractions. Synthetic prostaglandin E2 mimics the natural process of cervical softening through collagen breakdown and movement of an inflammatory infiltrate into the cervix [18]. Synthetic oxytocin is chemically identical to the endogenous form and stimulates uterine contractions when administered continuously by intravenous infusion [19], while the endogenous oxytocin is released in pulsatile manner. 

We hypothesized a possible interference on the placental oxygenation by “artificial” contraction, resulting in a modification of the autophagy expression. In our population, cases with induction of labor had achieved the neonatal birth after a longer time to vaginal delivery. It is known that, during labor, blood flow to the intervillous space is intermittent due to the interruption of the diastolic flow of the spiral arteries at the pick of the uterine contraction [20]. In placentas obtained after labor, many markers of injury from hypoxia reoxygenation have been detected, suggesting the presence of an oxidative stress [21–23], and autophagy can be induced by oxidative stress [24]. Our *a priori* hypothesis was that induction of labor can increase autophagy in placenta; actually we did not observe significant differences either in LC3 localization or LC3-II expression between cases with spontaneous labor and cases induced with synthetic oxytocin and/or prostaglandin; no significant relationship was also found between LC3-II and the time to vaginal delivery. Moreover, we observed similar pO_2_ and pCO_2_ levels in umbilical artery between groups; consequently, no differences in HIF-1*α* expression were found according to the mode of onset of labor. Hypoxia stabilizes HIF-1*α*, and HIF-1*α* is a major regulator of the cellular response to hypoxia [10]; moreover, hypoxia can induce autophagy [25–28]. Therefore, we speculated that similar levels of oxidative stress and hypoxia can be present in spontaneous and pharmacologically induced labor, although this hypothesis needs to be confirmed.

An interesting result of our study was to find a tight inverse relationship between LC3-II expression and the maternal pre-pregnancy body mass index: as BMI increased, placental autophagy decreased, irrespective of the mode of labor onset. A possible explanation of this finding might reside in the nutritional maternal habits before and throughout pregnancy rather than in rapid metabolic changes during labor, since we found no difference in umbilical arterial glucose concentrations or in acid-base equilibrium in the groups of women.

In conclusion, this is the first study assessing the influence of the mode of the onset of labor on placental expression of LC3. Our results suggest that autophagy expression is unaffected by the pharmacological induction of labor.

## Figures and Tables

**Table 1:** Clinical characteristics and autophagy.

	Spontaneous labor (*n* = 7)	Induced labor (*n* = 21)
Maternal age (years)	29.7 ± 5.8	31.9 ± 5.3
Gestational age (weeks)	39.0 ± 0.8	39.9 ± 1.2
Maternal pre-pregnancy BMI (kg/m^2^)	22.6 ± 3.7	22.5 ± 2.9
Neonatal birth weight (grams)	3271.4 ± 499.3	3412.0 ± 354.6
Placental weight (grams)	434.6 ± 90.1	441.3 ± 78.7
F/P ratio	7.6 ± 1.1	7.9 ± 1.3
Placental surface (mm^2^)	199.2 ± 55.9	219.1 ± 58.8
Time to parturition (minutes)	276.7 ± 499.3	660.2 ± 388.5*
pH	7.25 ± 0.09	7.25 ± 0.03
BE (mmol/L)	−5.4 ± 2.8	−4.3 ± 2.5
Lac (mmol/L)	6.1 ± 2.7	5.2 ± 1.8
pO_2_ (mmHg)	25.4 ± 5.5	20.5 ± 6.8
pCO_2_ (mmHg)	50.4 ± 11.8	51.1 ± 6.4
Glu (mmol/L)	4.9 ± 0.9	5.1 ± 1.2
LC3-II/*α*-tubulin (ODu × mm^2^)	1.4 ± 0.3	1.1 ± 0.1

BMI: body mass index; F/P ratio: ratio between fetal weight at birth and placental weight; BE: base excessl Lac: lactate; Glu: glucose; ODu: optical density unit.

*P = 0.03 versus spontaneous labor.
